# Medical Reform

**Published:** 1846-04-01

**Authors:** 


### EDITORIAL, MEDICAL INTELLIGENCE, &c.

*Medical Reform.* The improvement of medical instruction, the elevation of the standard of requirements for graduation, and the equalization of the qualifications for diplomas or other credentials of the regular practitioner, are, professedly, the most prominent of the subjects which will come before the National Convention. There seems to be entire unanimity in the profession respecting the necessity of reform in these several particulars. We apprehend that every reader of our journal would consider it gratuitous to argue this point. The present state of medical education is defective and insufficient. The attainments required to enter the ranks of the profession are inadequate, and the provisions regulating medical practice lack that uniformity in the different States which is desirable, and, considering the constant interchanges of population, highly important. The consequences of such a state of affairs are too obvious and familiar to require illustration. They are seen and felt by every intelligent and honorable member of the profession. We will not now prosecute an inquiry into the causes which have led to the evils to which we refer. On this topic difference of opinion probably exists. We think that erroneous views prevail to a considerable extent respecting it, and that undeserved blame is frequently attached to medical schools from an imaginary agency which they are supposed to exert in bringing about the results referred to. The true explanation we suppose to be briefly this : The regulation of the whole matter has been heretofore in the hands of legislation. It has been supposed to involve the public welfare and interests sufficiently to make it proper and necessary for the public to assume its control. Public sentiment, however, for many years has been opposed to this connection. Under the influence of a popular jealousy of all exclusive privileges, legal restrictions, always few and inefficient, have been withdrawn, the policy of legislative protection has been abandoned, until, at length, the disseverance is really or virtually complete throughout the union. The character and requisitions of the profession are in fact relinquished into the hands of its members. What the profession is to be for some time to come, is for the profession itself to decide. No concerted deliberation and action have as yet taken place. But the time has arrived when they are called for, and, it is to be hoped, with an earnestness and unanimity which will ensure important results. In the relations which legislation has assumed toward us, as respects any peculiar legal privileges, we do not, for one, complain ; but we think that the public welfare, not less than the character of the profession, has been overlooked in the determination to eradicate every vestige of what has treated as a remnant of aristocratic institutions. We would not ask an iota of legal protection either for our interests or honor : but in consideration of the public benefits which flow from the progressive advancement of medical science, and the importance to the public of well educated practitioners of medicine and surgery, we may with great propriety claim in behalf of our profession a more liberal legislative policy than is at present in vogue. We are willing to rely upon our own efforts to maintain, as individuals, the dignity and usefulness of the profession, and to trust to our abilities and assiduity for support, but, assuredly, it is not asking too much from legislation to provide facilities and encouragements for the promotion of knowledge and scientific progress. Let the State undertake, if it will, to educate all who wish to become practitioners at the public expense ; we will not complain that such a course would crowd the ranks of the profession ; only let the advantages and educational arrangements be of a high and worthy character—but we must defer farther considerations upon this topic. Our purpose in this article is to submit, briefly, our views of reform relating to improvements in medical instruction, and an uniform standard of acquirement. We proceed at once to this department of the subject.

The absence of any provision for proper preliminary education is one of the fundamental evils of the present system. A large proportion of the young men who enter upon the study of medicine are very imperfectly prepared by previous discipline and acquirement. Persons who would not be admitted as freshmen at any of our collegiate institutions, do not hesitate to enroll themselves as medical pupils; and, yet, how absurd to suppose that as much previous qualification, to say the least, as is necessary to commence a college course is not requisite for the novitiate in medical science! Instances have doubtless occured in which medical students sadly deficient in preliminary attainments, by dint of industry and perseverence during the period of medical pupilage and afterward, have made up for the loss of early advantages. There have been those, also, who by the force of superior talents have accomplished an enviable position in the profession without these qualifications. These, however, are exceptional cases, and do not affect the general rule. If a medical student be miserably deficient in mental training, and in the elementary branches of a good general education, the probability is he will make an unworthy member of the profession; and the fact that at this moment the profession embraces so many who are grossly ignorant in every department of a common education, and notoriously deficient in general intelligence, more than any thing else contributes to create dissatisfaction and disgust with the profession among well educated and intelligent minds. It is surely needless to amplify upon this point; nor is there room for difference of sentiment as to the minimum which should be required of the medical student before being permitted to enter upon the study. To a good proficiency in all the branches of what is called a common English education, should be added classical attainments sufficient, at least, for the philology of scientific nomenclatures; an acquaintance with the elements of Physics, Chemistry, and Natural History; and a fair knowledge of mental philosophy, embodying the fundamental principles of logic. It would be more satisfactory if evidence of these preliminary qualifications were furnished before the student actually enters upon medical studies. If medical schools would make it a rule to examine on this point before delivering tickets, the object might in this way be secured. But if this be not advisable, the diploma, or licence should never be bestowed without this evidence, together with proper testimonials of the intellectual and moral character of the applicant.

Next, as to Medical Studies proper. The advantages of oral instruction are too apparent, and have been too long recognised to require advocacy. But it must be conceded by all who have bestowed any reflection on the subject, that these advantages are very imperfectly enjoyed by the American student, owing to the shortness of the lecture terms in all of our medical institutions. It is usual for from five to seven lectures to be delivered daily during the term, and even then, in some, if not most of the departments, the courses are unfinished for want of time. In addition to this, it is generally expected that dissections and clinical observations will receive a considerable share of attention. Now, under these circumstances, no student can avail himself faithfully of all the advantages placed before him. It is impossible for the mind to receive, arrange and assimilate the ideas upon different subjects to which the attention is directed in such rapid succession. Passing from one lecture room to another, the facts presented in each succeeding hour supplant a large portion of those which have previously been received; the mental faculties become fatigued with the prolonged exercise of the attention, and the variety of ideas; and the consequence is, that if the pupil be ever so diligent and constant in his attendance, he fails to do justice both to himself, and to the efforts of his teachers. The most common result is, that after a fruitless effort to be attentive to all branches, he finds it absolutely necessary to neglect some, in order to improve the remainder.

We think that the lecture term should be extended, at least to six months. At most, but four lectures should be given daily. And it seems to us that it would be an improvement in all cases to classify the several departments, and have the attention of the student directed at one time to two or three only. Thus Anatomy, Physiology, and Surgery, are united by intimate relations, and may with great advantage be prosecuted simultaneously. Medical Chemistry, Pathology, Practical Medicine, and Materia Medica, have especial affinities. Midwifery and Legal Medicine, are frequently associated in the same chair. These details, however, do not belong to the present occasion.

Dissections should always be prosecuted out of the lecture term. Provisions are made for this at several of our colleges, but we fear they are not sufficiently embraced by students. If the student endeavors to carry on his labors in this province while he is attending lectures, it will surely follow that either *it*, or the lectures are neglected. It seems to us that it would be good policy to leave practical anatomy to private enterprise, requiring of the pupil as a qualification for graduation, proper evidence that he has pursued dissections to an extent deemed necessary. This would build up in all suitable localities, good private anatomical schools, and would greatly conduce to the increase of facilities for prosecuting this branch faithfully and thoroughly.

The same necessity also exists for constituting clinical medicine a branch of study, to be pursued out of lecture term. The introduction of cases to illustrate principles of medical and surgical practice in connexion with didactic teaching, is, of course, desirable; but to watch closely from day to day the progress of disease, and the effects of remedies, and connect suitable reading and reflection upon the pathological and therapeutical principles involved, will require too large a share of the student's attention to be divided with daily attendance upon several lectures—unless they are clinical lectures given in connection with the cases which are under observation. In proof that this position is correct, we would adduce facts which have lately been published by the editors of the New-York Journal of Medicine, and the Bulletin of Medical Science, relative to the actual attendance of the medical students who congregate in such large numbers during the lecture terms at New-York and Philadelphia. In New-York, out of six hundred students who were attending medical lectures at the two schools in that city, only twenty had taken the hospital ticket on the 22d Dec. last! In Philadelphia more than a thousand pupils were assembled the past winter at the three schools, and, of these, *ninety* took the ticket admitting to the Pennsylvania hospital! These facts speak sufficiently for themselves. Are students disinclined to clinical study? Certainly not; but if they give tolerable attendance to the lectures, they have neither time nor physical and mental strength for more.

The importance of clinical instruction is not sufficiently recognised in our present system. Didactic teaching can never qualify a student to enter at once upon the responsibilities of practice. If he do venture upon them with this preparation only, (as is often the case) he acquires that amount of practical observation and experience which is necessary to render him in any respect a safe practitioner, at the expense of the patients who trust their lives to his care in the early part of his career. This is the plain state of the case, and the appeal may very confidently be made to the personal retrospections of many of our readers for its confirmation. Medical schools should therefore require as a qualification for graduation, evidence of proper acquirements in Clinical medicine. This is the case in foreign schools, and it is one of the most serious defects of our system, that it is not in this country.

In order to meet the wants of the pupil, every hospital and alms-house, should be a school for instruction at the bed side, where students can become familiar with the phenomena of disease, and all the details of treatment, before they assume the responsibility of managing cases for themselves. Clinical lectures should be given at these institutions irrespective of the lecture session so called. This plan would also have the advantage of affording additional encouragement for medical abilities, and a new avenue for honorable distinction : it would develope talent, and prepare for the exercise of the more formal and comprehensive duties of the teacher.

To complete the course of study as we have briefly indicated, and to prepare the student to commence the duties of the practitioner, we conceive the present period of pupilage too short. We advocate its extension at least one year, and we should be better satisfied to have it fixed at five years.

Finally, (as we must bring this article to a close,) the occupation of the student out of his lecture term, should be better provided for than it generally is at present. We have already spoken of important branches which are to be pursued at these intervals. But in addition we deem the establishment of private schools at all favorable points, a measure of great importance. Situated as a large proportion of students are in the offices of private Physicians, without modern books, and with few or none of the functions of an instructor exercised in his behalf, his time is in a great measure lost. It is often worse than lost, for it frequently happens that he imbibes the routine errors of the practitioner, whose dogmatical maxims constitute the only lessons he receives, which, if he would afterward progress, he is compelled to unlearn. Without enlarging on this subject, we would refer our readers to some excellent remarks by Dr. Davis, in the January number of the New-York Journal of Medicine.

The number of the same valuable Journal for March, contains an address by Dr. F. Campbell Stewart, on the present condition of the medical profession, which embraces sound and judicious views, and commends itself to the perusal of all who take an interest in a matter, which at the present time, is invested with vital importance.

Since this article was penned we have received a copy of Dr. Stewart's address, which has been published in a pamphlet form. We trust it will be extensively circulated.

